# Investigating the role of family members in postnatal care: Evidence from mother-caregiver dyads in India

**DOI:** 10.1371/journal.pone.0327986

**Published:** 2025-09-10

**Authors:** Pooja Suri, Sahana SD, Shirley Yan, Seema Murthy, Jamie Sewan Johnston

**Affiliations:** 1 School of Public Health, University of California, Berkeley, California, United States of America; 2 YosAid Innovation Foundation, Bengaluru, Karnataka, India; 3 Noora Health, San Francisco, California, United States of America; 4 Noora Health India Private Limited, Bengaluru, Karnataka, India; 5 Stanford Center for Health Education, Stanford, California, United States of America; India Health Action Trust (IHAT), Uttar Pradesh Technical Support Unit (UP-TSU), INDIA

## Abstract

**Objectives:**

In this study, we examine the dynamics of birthing women relative to other family members in making caregiving decisions about postpartum maternal and infant care in four states in India. Specifically, we investigate the involvement of the father, maternal grandmother, and paternal grandmother of the newborn in household health decision-making.

**Methods:**

We analyze data from 551 dyads of women with infants under six months and the family caregiver identified as providing the primary support in the postpartum period. We present descriptive statistics on 1) the identity of the primary decision-maker as independently reported by birthing women and caregivers and 2) how disagreements are resolved. Within each dyad, we examine the level of agreement on health decision-making between mothers and caregivers. We use regression models to analyze the association between caregiver identity, and postpartum outcomes.

**Results:**

Our findings show that decisions in the household are predominantly made by a single person (around 70 percent), either the birthing woman or one of the caregivers – the father, maternal grandmother, or paternal grandmother of the newborn. Across all dyads, birthing women are more likely than other household members to name their caregivers as the sole decision-makers for infant care and their own. The involvement of birthing women in household decision-making is low, with less than a third of birthing women reporting involvement in either. Within-dyad agreement on the identity of sole decision-makers is low, with less than 30 percent of dyads in agreement for both infant and maternal care decision-making. Birthing women experience a higher level of mental well-being on a normalized index by 0.12 standard deviations (sd) when their primary caregiver is their own mother. In contrast, the mental well-being of the birthing women is negatively impacted by 0.10 sd when the caregiver is the mother-in-law. We also observe that the type of caregiver significantly impacts postpartum recovery of the birthing woman. Specifically, birthing women whose caregivers are mothers-in-law are 16.2 percentage points less likely to be well post-delivery. These findings enhance our understanding of the gendered role of caregivers in postnatal care in India, providing new insights into how caregiving and decision-making responsibilities are distributed within families.

## Introduction

The postpartum period is a critical time for the physical and mental recovery of the mother, as well as the optimal development of the newborn [1–3]. A significant proportion of deaths and re-hospitalizations for both mothers and newborns occur in the first few weeks following birth [4,5]. The Lancet’s neonatal survival series highlights practical and cost-effective interventions that improve maternal and neonatal health outcomes by enhancing maternal nutrition, promoting breastfeeding, and ensuring adequate and timely neonatal care [4,6–8]. However, these interventions often require considerable time and resources, which are frequently limited and pose implementation challenges [6,9,10]. Previous literature, such as the systematic reviews conducted by Martin et al. in 2020 and 2021, highlight the positive impact of family and caregiver support on maternal health outcomes [11,12]. Other studies have demonstrated the association between the involvement of family members who are also caregivers, and improved maternal mental health [13–16], maternal-infant bonding [17–19], and increased uptake of breastfeeding [20,21].

In India, the postpartum period is characterized by limited access to health information, and unique cultural context where women are considered vulnerable and reliant on other family members, especially their mothers and mothers-in-law for support [22,23]. Family caregivers in India greatly influence the mothers’ decisions during their pregnancy and the postpartum period; evidence suggests that the stronger the relationship quality, the more likely the mother is to engage in WHO-recommended best practices [24–27]. Nevertheless, the influence of different types of caregivers, especially mothers and mothers-in-law of the birthing woman, on health outcomes for both the mother and newborn remains poorly understood [28–30]. Additionally, the potential discordance between caregivers and mothers concerning postpartum care decisions adds further complexity to the caregiving dynamic [25,30,31]. Examining these intricate relationships is crucial for designing effective interventions and ensuring optimal postpartum health outcomes, particularly as maternal and infant mortality remains high in India [32]. Our study contributes to this gap in the literature by providing new insights into the distinct roles and influences of various family caregivers, such as mothers and mothers-in-law, in postpartum decision-making. By highlighting the often-overlooked decision-making authority of family members, our research underscores the importance of considering these dynamics when designing interventions aimed at improving maternal and infant health outcomes in India.

Several studies have examined the critical role of family caregivers in low- and middle-income countries (LMICs), particularly in South Asia, where mothers-in-law and other senior family members significantly influence postnatal care decisions, including breastfeeding practices and maternal nutrition [4–6]. Recent research has focused on the relationship with the mother-in-law in particular. Co-residence with in-laws can have both positive and negative effects. For example, having a mother-in-law in the household can enhance labor force participation [33], reduce the risk of iron-deficient anemia [34], and improve antenatal care visits and postnatal mental health through better daughter-in-law–mother-in-law relationships [22,35,36]. However, other studies suggest that co-residence, particularly with a mother-in-law, may diminish a woman’s authority in making her own health or labor participation decisions [22,23,33,34,37]. These findings highlight the complexity of caregiving dynamics and their impact on maternal health outcomes, which our study seeks to further explore.

Several barriers can impede caregivers’ ability to support mothers effectively in the postpartum period; limited knowledge, inadequate resources, and competing responsibilities often hinder caregivers from providing comprehensive care [26,38]. Additionally, cultural beliefs, social norms, and economic constraints can further contribute to suboptimal postpartum care practices [39,40]. This study explores the dynamics within birthing women and caregiver dyads, investigating the role of caregivers (primarily the father, maternal, and paternal grandmother of the newborn) and their influence on postpartum care in four states in India. In this study, we explore three key research questions: 1) How do different family caregivers influence household health decision-making, particularly in determining responsibilities for the mother’s health versus the infant’s health? 2) How are conflicts regarding decision-making resolved within the household? 3) How do these caregiver relationships impact postpartum health outcomes, including mental well-being? We hypothesize that 1) decisions regarding the infant’s health are made by multiple members of the household, and the type of family caregiver during the postpartum period is crucial. We expect that women cared for by their mothers-in-law will have poorer health outcomes compared to those cared for by their own mothers or spouses.

## Materials and methods

### Study design and participants

In this study, we present findings from a cohort of women who had recently given birth in hospitals where the non-profit organization Noora Health, through its local partner organizations, implements its neonatal Care Companion Program (CCP), a health education program that provides in-hospital training sessions and digital messaging on postnatal care to birthing women and their family caregivers. Noora Health aims to make family caregiver education part of the standard of care to strengthen postnatal care quality in the home and improve maternal and neonatal health outcomes. Additional details on CCP can be found in Yan et al. (2023). CCP is integrated into all district hospitals and specific medical colleges in the states included in this study (Karnataka, Madhya Pradesh, Maharashtra, and Punjab). All birthing women and family caregivers present in the postnatal wards during CCP training are invited to participate.

**Fig 1:**
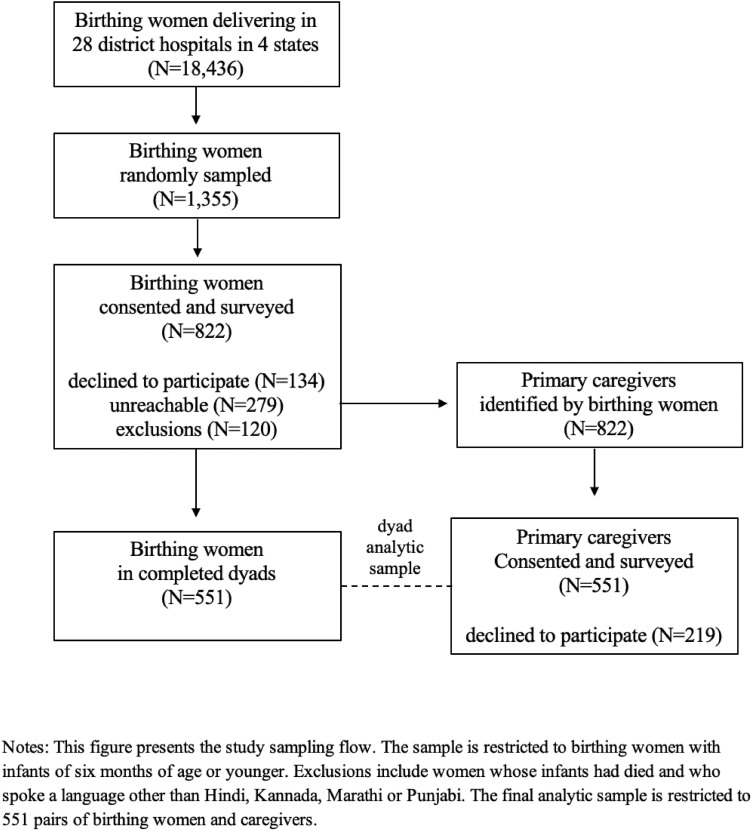
Study flow diagram.

Fig 1 shows the study sampling flow. The sample of birthing women in the study was randomly drawn from a larger random sample of high and low-delivery load hospitals of 18,436 birthing women who delivered in 28 district hospitals in the four states between September 2018 and May 2020 and completed a post-discharge survey conducted by Noora Health within one month after birth. Additional details on this sample can be found in Subramanian et al. (2020). Our sample was restricted to birthing women with infants six months of age or younger (born after December 2019), who were then stratified our sample by state and whether the infant fell sick at home post-discharge. These variables were selected as there are significant socioeconomic differences by state, and postpartum experience can be influenced by the infant’s health post-discharge. Each strata was sampled proportionate to the size of the hospital so that the unweighted analysis is population representative of eligible birthing women in the hospitals. Given our limited ability to determine the sample size, we did not conduct power calculations in advance. Birthing women whose infants had died and who spoke a language other than Hindi, Kannada, Marathi, or Punjabi were excluded from the sample due to ethical reasons and the unavailability of qualified survey staff with proficiency in languages other than the ones mentioned above. In total, we sampled 1,355 birthing women. The available budget and surveying capacity of the Noora Health field team determined this sample size. We recruited participants via phone between May and June of 2020. Of the total sample recruited, 822 birthing women were successfully contacted and consented to participate.

We first administered a 30-minute phone survey to consenting birthing women. At the end of the survey, we asked birthing women to provide their primary postpartum caregiver’s name and contact details. We then contacted and independently surveyed this caregiver. The survey restricted birthing women to name only one primary caregiver, and if they named more than one, they were asked to rank who is primary. A total of 551 caregivers consented to participate in the study. Because this study aims to analyze the birthing woman and caregiver dyads, we restrict our analytic sample to the 551 pairs of birthing women and caregivers for whom we have complete dyad data.

### Data collection

Our study draws upon two sources of data. We pooled phone survey data collected during recruitment for this study, with post-discharge data collected by Noora Health within 30 days after birth for the same women in our study [41]. Post-discharge data collected from birthing women includes information on the age of the birthing woman; data on prior pregnancies of the birthing woman, including the number of times they have been pregnant, whether this is their first child, and number of existing children; data on postpartum practices, including exclusive breastfeeding, skin-to-skin care, umbilical cord care, and maternal diet restrictions; and health outcomes including hospital readmissions of infants and birthing women and mental health of birthing women.

Phone surveys administered to birthing women collected household demographic data, including household size, rural residence, and socioeconomic status measured by an asset score and below-poverty line status; health knowledge as proxied by a series of questions asked about COVID-19 awareness and prevention due to the timing of the survey coinciding with the start of the COVID-19 pandemic; family caregiver support and intra-household decision making about maternal and infant care; and maternal mental well-being.

The phone survey administered to caregivers included the same questions as the birthing woman survey, except for questions about mental well-being.

### Measures

We describe the types of family caregivers that provide postpartum support to birthing women. While the birthing women reported many different family relationship types among their primary caregivers, we limit our primary analyses to the three most frequently mentioned relationship types: 1) the spouse of the birthing woman, 2) the mother of the birthing woman, and 3) the mother-in-law of the birthing woman. We included caregivers, who were mentioned by more than 10 percent of the sample, which led us to focus on these three groups. In addition, we focus specifically on this because of the growing body of work on these relationships [42–45]. These three relationship types comprise 74.05 percent of family caregivers identified (see S1 Table in S1 File). Other commonly mentioned relationship types include sister, sister-in-law, daughter, and father.

**Fig 2:**
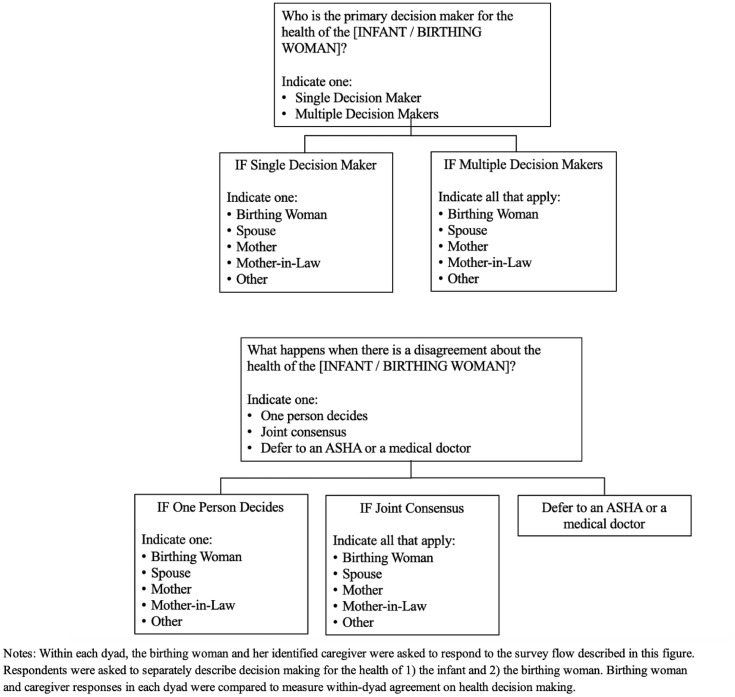
Health decision-making survey flow.

We leverage data collected from family caregivers to examine the level of agreement regarding health decision-making within the birthing women and caregiver dyads. Fig 2 describes the flow of survey questions investigating health decision-making for the infant and separately for the birthing woman. Each dyad member was asked to identify 1) the identity of the primary decision maker(s) (single or multiple) and 2) how disagreements are resolved (by a single decision maker, joint consensus, or by deferring to the advice of an ASHA or medical doctor). Within each dyad, the birthing woman’s and the caregiver’s responses were compared to understand the within-dyad agreement.

Finally, we examine the association between family caregiver type, postpartum care practices, and maternal mental well-being. The postpartum practices that we examine include the following indicators: 1) exclusive breastfeeding, 2) practice of skin-to-skin care, 3) unsafe umbilical cord care (i.e., application of anything to the cord), 4) restriction of the birthing woman’s diet, 5) if the infant fell sick post-discharge, 6) if the birthing woman fell sick post-discharge, and 7) missed vaccinations for the infant. We constructed a maternal mental well-being index from a series of yes/ no statements asking birthing women about their anxieties, caregiving load, stress, and available support. We constructed the index by taking the unweighted sum with higher values indicating poorer mental well-being and standardizing around the mean for ease of interpretation. This is a novel index, and has not been validated elsewhere.

### Statistical analysis

All statistical analyses were conducted in Stata 16 and Stata 18. To estimate the association between the identity of the primary caregiver and the various health outcomes, we use logistic regression when the dependent variables are binary and Ordinary Least Squares (OLS) when the dependent variable is continuous.

Our main estimating equation presented is a logistic regression:


$$log\;(P_{ih})\;=\;\alpha \;+\;\beta 1\;C_{h}\;+\;\gamma \;X_{ihc}\;+\;\theta_{sh}\;+\;\varepsilon_{ih}$$ (1)

where the dependent variable *P*_*ih*_ represents the probability of an outcome for a mother *i* recruited in hospital *h*. The variable *C*_*h*_ is a categorical variable, which is 0 if the caregiver is the spouse, 1 if the caregiver is the mother, and 2 if the caregiver is the mother-in-law. We excluded the ‘Other caregivers’ category from our analysis, as it is not possible to consolidate them into a single relationship type. The vector *X*_*ihc*_ consists of mother, caregiver and hospital-level covariates such as the age of the birthing woman and the caregiver, household size, number of existing children, and parity, as well as indicators for rural residence and below-poverty line status. Additionally, we include an asset score, where a higher score indicates better socioeconomic status, and a health knowledge index constructed as a standardized unweighted sum of correct responses to questions related to COVID-19. We also include state-and-hospital level fixed effects (*θ*_*sh*_). We use a logistic regression model to estimate the results as outcomes are binary and report risk differences (RD) using the Stata *margins* command. We checked for multicollinearity using variance inflation factors (VIF < 5) and used robust standard errors to address heteroscedasticity concerns in our models. We include state-fixed effects to hold constant state-wise variations.

### Ethics review

This study received ethics approval from the Institutional Review Board (IRB) at Stanford University, California (protocol number IRB-55003), and the ACE Ethics Committee based in Bangalore, India. At the start of each survey, a consent script was read to participants in their local language, and participants were asked to provide verbal consent. Only those who provided consent were included in the study. Participants were not compensated for their time financially or otherwise. The primary data with identifiable information were collected and maintained in India; de-identified data were shared with collaborators for analysis.

## Results

We begin the discussion of our findings with Tables 1 and 2, which describes the birthing women included in our sample, and their primary caregivers comprising the dyads. Subsequently, in Table 3, we present a descriptive analysis of household decision-making within the dyads. Finally, in Table 4, we describe the association between the postpartum outcomes of the birthing woman and the type of primary caregiver.

**Table 1:** Birthing woman descriptive characteristics.

	Birthing woman (n = 551)
Age	24.14 ± 3.66
Live in a rural village	368 (66.79)
State	
Madhya Pradesh	176 (31.94)
Karnataka	254 (46.10)
Punjab	69 (12.52)
Maharashtra	52 (9.44)
Caste	
General	162 (36.00)
Scheduled caste (SC)	107 (23.78)
Scheduled tribe (ST)	56 (12.44)
Not specified	226 (41.02)
Primary spoken language	
Hindi	285 (51.72)
Kannada	177 (32.12)
Punjabi	43 (7.80)
Marathi	46 (8.35)
Socio-economic status	
Ownership of HH goods – out of 7	4.86 ± 0.05
Ownership of Below Poverty Line (BPL) Card	383 (69.51)
Household characteristics	
Household size	4.50 ± 0.07
Number of years married	4.64 ± 0.15
Number of previous children	1.27 ± 0.03
Birth-related characteristics	
First-time mothers	299 (54.36)
Postpartum residence in maternal home	385 (69.87)
COVID-19 Health knowledge index – score out of 15	3.94 ± 2.03

Notes: This table presents the descriptive characteristics of birthing women in the analysis sample. Data are displayed as either mean±SD or n (%). Socio-economic status is presented as a score by counting the ownership of seven common household goods. Ownership of the Below Poverty Line (BPL) card is an indicator for whether the respondent’s family has a government issued card. Postpartum residence in maternal home is an indicator for whether the respondent (birthing woman) lived in their mother’s home immediately following birth. The health knowledge index is constructed by taking the sum of the respondent’s score on a 6 knowledge questions relating to COVID-19.

**Table 2:** Primary caregiver characteristics.

	All dyads: Caregiver survey (n = 551)			
	Spouse (n = 170)	Mother (n = 142)	Mother-in-law (n = 96)	Other caregivers (n = 143)
Age	30.02 ± 6.79	43.62 ± 7.51	48.75 ± 7.52	37.90 ± 13.35
Live in a rural village	110 (64.71)	115 (80.99)	60 (62.5)	95 (66.43)
Highest level of education completed				
Primary school completed	25 (14.71)	91 (64.08)	61 (63.54)	43 (30.07)
Less than secondary school	94 (55.29)	33 (23.24)	15 (15.62)	51 (35.66)
Secondary school completed	29 (17.06)	1 (0.70)	3 (3.12)	12 (8.39)
Higher than secondary school	18 (10.59)	0 (0)	1 (1.04)	15 (10.49)
Do not know	4 (2.35)	17 (11.97)	16 (16.67)	22 (15.38)
Health knowledge index – score out of 15	4.28 ± 0.15	2.92 ± 0.13	3.16 ± 0.20	3.80 ± 0.17

Notes: This table presents the descriptive characteristics of the caregivers in the analysis sample. Data are displayed as either mean±SD or n(%). We combine primary caregiver relationships that don’t include the spouse, mother, and mother-in-law as other caregivers. The health knowledge index is constructed by summing the respondent’s score on a 6 knowledge questions relating to COVID-19.

**Table 3:** Dyad decision making: infant care decisions.

	Core dyads: birthing woman survey (n = 408)			Core dyads: caregiver survey (n = 408)		
Primary caregiver	Spouse (n = 170)	Mother (n = 142)	Mother-in-law (n = 96)	Spouse (n = 170)	Mother (n = 142)	Mother-in-law (n = 96)
***Primary decision-maker for the baby***						
**Single decision-maker**	**137 (80.59)**	**107 (75.35)**	**72 (75.00)**	**124 (72.94)**	**118 (83.09)**	**77 (80.20)**
Birthing woman (self)	38 (27.74)	23 (21.50)	16 (22.22)	37 (29.83)	55 (46.61)	30 (38.96)
Spouse	55 (40.15)	9 (8.41)	7 (9.72)	56 (45.16)	9 (7.62)	5 (6.49)
Mother	17 (12.41)	65 (60.75)	10 (13.89)	15 (12.10)	41 (34.74)	11 (14.28)
Mother-in-law	20 (14.60)	3 (2.80)	33 (45.83)	5 (4.03)	1 (0.84)	24 (31.17)
Other	7 (5.11)	7 (6.54)	6 (8.33)	11 (8.87)	12 (10.16)	7(9.09)
Dyads agreed on identity of single decision-maker	44 (28.21)	26 (20.16)	24 (28.57)	–	–	–
**Multiple decision-makers**	**33 (19.41)**	**35 (24.65)**	**24 (25.00)**	**46 (27.06)**	**24 (16.90)**	**19 (19.79)**
Birthing woman involved	20 (60.61)	25 (71.43)	11 (45.83)	28 (60.87)	19 (79.17)	6 (31.58)
Spouse involved	24 (72.73)	4 (11.43)	12 (50.00)	28 (60.87)	3 (12.50)	7 (36.84)
Mother involved	5 (15.15)	28 (80.00)	9 (37.50)	12 (26.09)	5 (20.83)	7 (36.84)
Mother-in-law involved	9 (27.27)	1 (2.86)	15 (62.50)	5 (10.87)	1 (4.17)	14 (73.68)
Dyads with birthing women as a decision-maker	58 (34.12)	38 (33.80)	27 (28.13)	65 (38.24)	74 (52.11)	36 (37.50)
***Resolution of disagreements for decisions about the baby***						
**Single decision-maker**	**39 (22.94)**	**60 (42.25)**	**25 (26.04)**	**47 (27.64)**	**53 (37.32)**	**34 (35.42)**
Birthing woman (self)	12 (30.77)	15 (25.00)	3 (12.00)	25 (53.19)	31 (58.49)	21 (61.76)
Spouse	18 (46.15)	4 (6.67)	6 (24.00)	8 (17.02)	4 (7.55)	0 (0.00)
Mother	2 (5.13)	36 (60.00)	0 (0.00)	7 (14.89)	5 (9.43)	1 (2.94)
Mother-in-law	4 (10.26)	1 (1.67)	16 (64.00)	2 (4.25)	0 (0.00)	6 (17.65)
**Multiple decision-makers**	**81 (47.65)**	**30 (21.13)**	**38 (39.59)**	**74 (43.53)**	**38 (26.76)**	**34 (35.42)**
Birthing woman involved	26 (32.10)	8 (26.67)	18 (47.37)	30 (40.54)	28 (73.68)	19 (55.88)
Spouse involved	65 (80.25)	13 (43.33)	23 (60.53)	60 (81.08)	19 (50.00)	18 (52.94)
Mother involved	1 (1.23)	22 (73.33)	3 (7.89)	9 (12.16)	6 (15.79)	4 (11.76)
Mother-in-law involved	23 (28.40)	4 (13.33)	28 (73.68)	4 (5.41)	1 (2.63)	12 (35.29)

Notes: This table displays descriptive statistics related to decision-making for infant care. Data are displayed as n(%). The responses from birthing women are organized according to the dyad they identify with, allowing for comparison within dyad type responses. In cases of dyad agreement on a single decision-maker, this table excludes agreements marked as “Other” in the survey. Similarly, for the resolution of disagreements, it does not present data related to the two additional options: 1) asking a community health worker or doctor, and 2) other.

**Table 4:** Dyad decision making: maternal care decisions.

	Core dyads: birthing woman survey (n = 408)			Core dyads: caregiver survey (n = 408)		
Primary caregiver	Spouse (n = 170)	Mother (n = 142)	Mother in Law (n = 96)	Spouse (n = 170)	Mother (n = 142)	Mother in Law (n = 96)
***Primary decision-maker for the birthing woman***						
**Single decision-maker**	**138 (81.18)**	**107 (75.35)**	**76 (79.17)**	**133 (78.24)**	**126 (88.73)**	**75 (78.13)**
Birthing woman (self)	17 (12.32)	32 (29.91)	8 (8.33)	55 (41.35)	81 (64.29)	42 (56.00)
Spouse	110 (79.71)	**20 (18.69)**	**32 (33.33)**	60 (45.11)	11 (8.73)	5 (6.66)
Mother	1 (0.72)	46 (42.99)	2 (2.08)	8 (6.02)	24 (19.05)	2 (2.66)
Mother-in-law	6 (4.35)	2 (1.87)	30 (31.25)	4 (3.01)	1 (0.79)	16 (21.33)
Other	4 (2.90)	7 (6.54)	4 (4.17)	6 (4.51)	9 (7.14)	10 (13.33)
Dyads agreed on identity of single decision-maker	53 (32.92)	37 (27.41)	15 (17.05)	–	–	–
**Multiple decision-makers**	**32 (18.82)**	**35 (24.65)**	**20 (20.83)**	**37 (21.76)**	**16 (11.27)**	**21 (21.87)**
Birthing woman involved	13 (40.63)	8 (22.86)	5 (25.00)	28 (75.68)	11 (68.75)	10(47.62)
Spouse involved	22 (68.75)	11 (31.43)	15 (75.00)	25(67.57)	7 (43.75)	13(61.90)
Mother involved	1 (3.13)	29 (82.86)	1 (5.00)	11 (29.73)	4 (25.00)	2 (9.52)
Mother-in-law involved	17 (53.13)	4 (11.43)	17 (85.00)	7(18.92)	2 (12.50)	8(38.10)
Dyads with birthing women as a decision-maker	30 (17.65)	40 (28.17)	13 (13.54)	83 (48.82)	92 (64.79)	52 (54.17)
***Resolution of disagreements for decisions about the birthing woman***						
**Single decision-maker**	**55 (32.35)**	**62 (43.66)**	**38 (39.58)**	**55 (32.35)**	**60 (42.25)**	**30 (31.25)**
Birthing woman (self)	8 (14.55)	19 (30.65)	9 (23.68)	35 (63.64)	40 (66.67)	19 (63.33)
Spouse	43 (78.18)	12 (19.35)	9 (23.68)	11 (20.00)	5 (8.33)	1 (3.33)
Mother	0 (0.00)	29 (46.77)	2 (5.26)	4 (7.27)	2 (3.33)	1 (3.33)
Mother-in-law	3 (5.45)	1 (1.61)	17 (44.74)	2 (3.64)	1 (1.67)	2 (6.67)
**Multiple decision-makers**	**69 (40.59)**	**29 (20.42)**	**26 (27.08)**	**73 (42.94)**	**31 (21.83)**	**38 (39.58)**
Birthing woman involved	19 (27.54)	6 (20.69)	8 (30.77)	29 (39.73)	18 (58.06)	19 (50.00)
Spouse involved	67 (97.10)	12 (41.38)	20 (76.92)	62 (84.93)	17 (54.84)	18 (47.37)
Mother involved	2 (2.90)	19 (65.52)	1 (3.85)	12 (16.44)	5 (16.13)	5 (13.16)
Mother-in-law involved	17 (24.64)	2 (6.90)	19 (73.08)	6 (8.22)	2 (6.45)	15 (39.47)

Notes: This table displays descriptive statistics related to decision-making for maternal care. Data are displayed as n(%). The responses from birthing women are organized according to the dyad they identify with, allowing for comparison within dyad type responses. In cases of dyad agreement on a single decision-maker, this table excludes agreements marked as “Other” in the survey. Similarly, for the resolution of disagreements, it does not present data related to the two additional options: 1) asking a community health worker or doctor, and 2) other.

1) *Dyad description*

Table 1 presents the descriptive characteristics of the birthing women in our sample. The mean age of the women is 24.14 years (sd = 3.66). Most women live in rural villages (66.79 percent), and below the poverty line (69.51 percent), owning 4.86 out of 7 everyday household items on average. The average household size is 4.50. Women have been married for 4.64 years, and more than half (54.36 percent) are first-time mothers. On the health knowledge index, the women score low, with a mean score of 3.94 out of 15.

551 birthing women identified a primary caregiver for postpartum recovery and infant care. The most common caregivers include the birthing woman’s spouse (30.85 percent), mothers (25.78 percent), and mothers-in-law (17.42 percent). Other relationships mentioned include but are not limited to, the sister, sister-in-law, and the birthing woman’s father (see S1 Table in S1 File).

Table 2 presents the descriptive characteristics of the most common primary caregivers: the spouse, mother, and mother-in-law, along with a column that consolidates all other caregivers. We see that spouses are older than the birthing women, at an average age of 30.02 years. The mothers-in-law are average 48.75 years, compared to mothers with an average age of 43.62 years. As with the birthing women’s sample in Table 1, more than half of caregivers live in rural villages. Among these three types of caregivers, spouses tend to be more educated, with a higher percentage having completed secondary school and higher than secondary education. In contrast, most mothers and mothers-in-law have only completed primary education. Spouses score higher on the COVID-19 health knowledge index (4.28 out of 15) compared to mothers (2.92 out of 15) and mothers-in-law (3.16 out of 15).

Tables 3 and 4 describe health decision-making for 1) infant care and 2) maternal care. As shown in Fig 2, in each dyad, birthing women and caregivers were asked to identify the primary decision maker(s) and decision maker(s) when there was a disagreement in the household about the health of the infant or birthing woman. The tables also show the percentage of dyads in which both members agree on all questions. The analysis was restricted to core dyads (N = 408) where the caregiver was a spouse, mother, or mother-in-law.

2) *Infant care decision-making*
  2.1) *Primary decision-makers for infants*

Table 3 shows the results of decision-making for the infant’s health by dyad type. The majority of respondents indicated that a single person made decisions for the infant’s health. Birthing women were most likely to name their primary caregiver (dyad member), as the decision-maker; 40.15 percent in the spouse dyad, 60.75 percent in the mother dyad and 45.83 percent in the mother-in-law dyad. Across all dyads, women were less likely to name themselves as the single decision maker, with 27.74, 21.50, and 22.22 percent identifying themselves, respectively. Across all dyads, mothers and mothers-in-law are frequently identified as decision-makers.

Among caregivers naming a single decision maker, spouses (fathers of the infants) were more likely to name themselves (45.16 percent). In contrast, mothers and mothers-in-law were more likely to consider the birthing woman the sole decision-maker. After the birthing women, mothers, and mothers-in-law were most likely to identify themselves as sole decision-makers. Few mothers and mothers-in-law identified spouses as decision-makers.

In Table 3, we also show agreement on single decision-maker identity. Agreement is similar for the spouse and mother-in-law dyads (28.21 and 28.57 percent, respectively) and slightly lower for the mother dyad (20.16 percent).

Among responses indicating multiple decision-makers, we examine the involvement of the birthing woman (mother of the infant), the spouse (father of the infant), the mother (maternal grandmother), and the mother-in-law (paternal grandmother of the infant). More than half of birthing women in spouse and mother dyads are involved in decisions (60.61 percent and 71.43 percent). Fewer birthing women are involved in mother-in-law dyads (45.83 percent).

Among the caregiver responses, we see the proportion of birthing women involved in infant care decisions made jointly is similar to those reported by the birthing women. However, mothers-in-law report lower birthing woman involvement compared to birthing women within the mother-in-law dyad (31.58 vs. 45.83 percent). We also see that spouses and mothers-in-law report the spouse being involved, whereas the mothers of birthing women rarely include spouses. Both spouses and mothers do not consider mothers-in-law involved, but a 73.68 percent of mothers-in-law consider themselves involved.

Table 3 also shows birthing women’s involvement in decision-making, either as a single or joint decision-maker. Birthing women report being involved in a third of dyads (34.12 percent spouse, 33.8 percent mother, 28.13 percent mother-in-law). Spouses and mothers-in-law report slightly higher involvement, and mothers report the highest involvement.

2.2) *Resolution of infant-care disagreements*

Finally, Table 3 presents data on the resolution of disagreements related to health decision-making for the infant. Similar to the previous results, birthing women tend to defer the ultimate decision-making during disagreements to their dyad member: 46.15 percent in the spouse dyad, 60 percent in the mother dyad, and 64 percent in the mother-in-law dyad.

3) *Maternal care decision making*
  3.1) *Primary decision-makers for birthing women*

Table 4 shows decision-making for the birthing woman’s health. Similar to Table 3, we present the results for the birthing women and caregivers by core dyad. Most birthing women and caregivers indicated that a single person makes the maternal care decisions. Birthing women in spouse dyads were most likely to name their spouse as the decision-maker (79.71 percent). Birthing women in mother and mother-in-law dyads were also more likely to name the caregiver as the sole decision maker, although to a lesser degree. Across all types of core dyads, women were less likely to name themselves the single decision-maker for her own health, with 12.32, 29.91, and 8.33 percent identifying themselves in spouse, mother, and mother-in-law dyads, respectively. Across all dyad types, birthing women identify spouses as primary decision makers for maternal care decisions at a higher rate than for infant-care decisions.

Among caregivers, spouses named themselves as decision-makers (45.11 percent) nearly as often as they named the birthing woman (41.35 percent). Compared to spouses, mothers and mothers-in-law were more likely to consider the birthing woman the primary single decision-maker for her health, with 64.29 percent of mothers and 56.00 percent of mothers-in-law identifying the birthing woman. Few mothers and mothers-in-law indicated that spouses made decisions for the birthing woman’s health.

Table 4 also shows dyads’ agreement on the single decision-maker for maternal care. Agreement is higher in spouse (32.92 percent) and mother (27.41 percent) dyads, and lowest in mother-in-law dyads (17.05 percent).

Among responses indicating multiple decision-makers, we examine the involvement of the birthing woman and each caregiver. More birthing women report involvement in decision-making for their care in the spouse dyad (40.63 percent) compared to the mother and mother-in-law dyads (22.86 and 25.00 percent, respectively). Among the caregiver responses, we see the proportion of birthing women involved in decisions regarding their care is much higher than those reported by the birthing women (75.68, 68.75, and 47.62 percent, respectively). Spouses are likewise included as multiple decision-makers across all dyad types at similar rates.

Similar to Table 3, we show the proportion of times the birthing woman is included as a decision-maker for her health across single and household-level decision-making. Across all dyad types, birthing women are much less likely to report their involvement in decision-making compared to caregivers reporting the involvement of birthing women. Birthing women reported involvement in under a third of dyads across all types (17.65, 28.17, and 13.54 percent in spouse, mother, and mother-in-law dyads, respectively. Caregivers reported birthing women involvement in a majority of dyads across all types (48.82, 64.79, and 54.17 percent in spouse, mother, and mother-in-law dyads, respectively).

3.2) *Resolution of maternal-care disagreements*

Table 4 also presents data on resolving disagreements related to health decision-making for the birthing woman. Similar to the resolution of infant-care disagreements (Table 3), birthing women tend to defer the ultimate decision-making during disagreements to their dyad member (78.18 percent in spouse dyads, 46.77 percent in mother dyads, and 44.74 in mother-in-law dyads).

4) *Postpartum care and mental well-being by caregiver type*

Table 5 presents the estimated associations between the type of primary caregiver and postpartum care practices for birthing women. The analysis revealed several significant associations between postpartum outcomes and the type of primary caregiver. Notably, when the caregiver was the birthing woman’s own mother, her mental well-being was significantly better than the reference group which includes the spouse. On average, the mental well-being of the birthing woman was 0.12 sdhigher (p-value < 0.1). Conversely, the mental well-being of the birthing woman is significantly negatively associated with the caregiver being her mother-in-law, 0.10 sd lower on the mental well-being index (p-value < 0.1).

**Table 5:** Postpartum health outcomes of birthing woman by type of primary caregiver.

	(1)			(2)				
	Mother			Mother-in-law			Mean of reference group (Spouse)	
	RD	*p*-value		RD	*p*-value			N
Mental well-being index	0.122	0.085	*	−0.105	0.097	*	0.000	408
Practicing exclusive breastfeeding	0.096	0.109		0.084	0.169		0.794	408
Practicing skin-to-skin care	−0.086	0.541		−0.295	0.061	*	0.606	112
Practicing safe cord care	0.092	0.115		0.072	0.212		0.429	408
Practicing unrestricted dietary practics	−0.036	0.554		0.084	0.222		0.194	408
Infant is well post-discharge	−0.031	0.619		0.046	0.442		0.782	408
Birthing woman is well post-discharge	−0.052	0.392		−0.162	0.016	**	0.775	408
Infant’s vaccinations are current	−0.021	0.719		0.016	0.802		0.850	408

Notes: This table presents the results of a logistic regression of the primary outcomes by type of primary caregiver. Each row represents a separate regression. The maternal mental well-being index is a continuous outcome constructed from a series of yes/ no statements asking birthing women about their anxieties, caregiving load, stress, and available support. The index is cacluated by taking the unweighted sum with higher values indicating poorer mental well-being and standardizing around the mean of the reference group. All other dependent variables presented are indicators that are 0 if the behavior was not practiced, and 1 if the behavior was practiced. The independent variable is 0 if the spouse is the caregiver, 1 if the mother is the caregiver, and 2 if the mother-in-law is the caregiver.Covariates included in the model are mother’s age, caregiver’s age, household size, number of previous children, parity, rural residence, below poverty line status, asset score, and health knowledge score. Standard errors (not shown here) are robust, and we include state and hospital fixed efffects. * denotes statistical significant at 10 pct, ** at 5 pct, and *** at 1 pct level. RD: risk difference

We observed few significant associations between the type of caregiver and postpartum care practices. However, we did observe that having a mother-in-law as a caregiver is associated with a 0.16 percentage point (pp) decrease in birthing women being well post-discharge compare to the reference group of spouse (*p* < 0.05), and a 0.30 pp decrease in birthing women practicing skin-to-skin care compared to the reference group of spouse (*p* < 0.05),

## Discussion

In this study, we examine the role of family caregivers in India in the postpartum period, investigating which household members provide primary support to birthing women and how decisions are made about maternal and infant care. Our findings show that the three most common family members supporting birthing women are the birthing woman’s spouse, mother, and mother-in-law. Our study contributes to the literature by using birthing woman-caregiver dyad data to examine how birthing women and their family caregivers perceive how decisions are made and the extent to which they agree, offering a unique glimpse into the spousal and intergenerational dynamics of household decision-making.

In India, in particular, complex family structures like multigenerational households, or household with strong figures like the mother-in-law can play a vital role in providing care for children, the elderly, and perinatal support [22,23,34]. Family caregivers play a significant role in household decision-making, often acting as the sole decision-maker for questions about the care of both children, and birthing women [22,35,36]. However, there is limited evidence on how this affects decision-making in the household. In this study, we observe that in the majority of households, decisions are made by a single person. Across all dyad types, birthing women were more likely to name their caregiver as the sole decision maker, not just for infant care but also for decisions about their own health. Caregivers were likewise more likely to name themselves as the sole decision maker over the birthing woman, although at slightly lower rates. Households shift to increased joint decision-making to resolve disagreements. Nevertheless, family caregivers are involved in decision-making in nearly all households in the study.

Our study suggests that birthing women overwhelmingly do not view themselves as primary household decision-makers. For both infant and maternal care decision-making, the involvement of birthing women as a primary decision-maker is notably low, particularly from the perspective of birthing women themselves. Birthing women report involvement as a primary decision maker, either as a sole decision maker or as part of joint decision making, in roughly a third of the dyads observed for infant care decisions and under a third of dyads for maternal care decisions.

In contrast, caregivers do report a higher involvement of birthing women, with slightly higher rates for infant care, and substantially higher for maternal care (over 50 percent of dyads). This mismatch, with birthing women deferring decision-making to caregivers, is reflected in low levels of agreement within the dyads about the identity of sole decision-makers. Agreement on sole decision-making in under a third of dyads for both infant and maternal care. For both primary decision-making and resolution of disagreements, birthing women assigned more authority to their caregiver counterpart, while caregivers reported deferring more authority to birthing women. The presence of multiple family decision-makers in some households may also contribute to the observed discrepancies and complicate the classification of decision-making roles. While the categorization of decision-making into single or joint frameworks allows for measurable analysis, it simplifies the nature of household decision-making. Future research should explore more detailed models to better capture these complexities.

Our study also points to important distinctions regarding the identity of the family caregivers, with patterns diverging depending on whether the primary caregiver is the birthing woman’s spouse, own mother, or mother-in-law. We observe notable gender dynamics at play with respect to infant care. According to birthing women, spouses are far less likely to be involved with infant care decisions compared to decisions about maternal health, suggesting birthing women may feel more authority for infant care than for autonomy over their own care. Female caregivers (i.e., mothers and mothers-in-law) are unlikely to assign infant care decision-making authority to infants’ fathers. However, with limited self-reported data, we could not fully explore the underlying factors behind these differences. We also acknowledge the possibility of caregivers over-or under-reporting their involvement and influence, highlighting the need for further research on this.

Furthermore, the identity of caregivers appears to matter for the mental well-being of birthing women. Having one’s own mother as a primary caregiver is strongly positively associated with better mental well-being compared to other types of primary caregivers, while having a mother-in-law as a primary caregiver is negatively associated with mental well-being. We also note that birthing women’s mothers are more likely to assign decision-making authority to their daughters compared to birthing women’s spouses and mothers-in-law. While birthing women derive the most agency and mental well-being benefits from caregiving provided by their mothers, it is important to note that this support may be short-lived, as most women in India typically move back into their spouse’s family home shortly after childbirth [35].

Our study’s findings align with existing research from South Asia, where family caregivers, such as mothers-in-law, often play pivotal roles in postnatal care. Multigenerational households and strong family hierarchies, heavily influence decision-making around maternal and infant care [46–48]. Our results are consistent with studies that indicate women, especially birthing women, often have limited autonomy in health-related decisions, with older family members like mothers-in-law acting as primary decision-makers [35,36,49]. However, while much of the literature points to the authoritative role of mothers-in-law, our study highlights that birthing women’s own mothers tend to provide greater agency and support, contributing positively to their mental well-being. While existing studies discuss broader caregiving dynamics, our study adds new insights by revealing nuanced differences in how spousal, maternal, and in-law caregivers impact decision-making and maternal agency during the postpartum period.

This study is the first to examine this household dynamic by studying family member dyads and decision-making, especially around health decision-making. However, the study has several limitations: 1) we use self-reported data may introduce bias, and impact the accuracy of our findings; 2) the mental well-being index, constructed from self-reported yes/no statements was used for ease of data collection over the phone may not capture the complexity of mental health for the population; 3) the sample was contacted over the phone during the COVID-19 pandemic, excluding individuals with limited phone access or time to respond, which may affect the generalizability of the results. Finally, the data was collected at the start of the COVID-19 pandemic, and this may have influenced family dynamics, including potential shifts in caregiving roles or decision-making responsibilities due to lockdowns or illness. Additionally, reduced access to healthcare services during this period could have affected participants’ experiences with maternal and infant care decisions. Lastly, we could be seeing increased mental health stressors brought on by the pandemic.

Our study lacks an experimental design, and has a small sample size which limits our ability our ability to fully analyze the extent of these dynamics’ impact on mental health and additional postpartum outcomes. Future research should study the effect of this dynamic on health decision-making, including health-seeking behavior, and the factors that influence how different household members respond to health information, and their access to it.

Our findings underscore the significant role family members play in postpartum decision-making, often acting as the sole decision-makers for both infant and maternal care. Notably, these decisions are frequently made by a single household member, who is often not the birthing woman. These insights have important implications for health policymakers and the design of programs aimed at improving maternal-child care in the postpartum period. Current interventions often employ a direct-to-beneficiary approach, overlooking the critical influence of family members on healthcare decisions [50]. We suggest that policymakers adopt a family-centered approach to healthcare, ensuring that information and support are directed to those who actually make household decisions, in contrast to the prevalent direct-to-beneficiary design that neglects the essential role of family in health-seeking behaviors and outcomes.

As in India, households in many LMICs are predominantly intergenerational [36]. Our research underscores the importance of designing interventions sensitive to these nuances, acknowledging that family caregivers must be incorporated into health education and can be instrumental in improving postpartum health outcomes. Our study contributes to the field by highlighting the critical influence of family caregivers on postpartum decision-making in India, emphasizing the need for culturally tailored interventions that account for the caregiving dynamics in multigenerational households, with future research needed to explore how these dynamics impact maternal autonomy and health outcomes across similar contexts.

## Supporting information

S1 File S1 and S2 Tables. (DOCX)
