# Correction: Investigating the role of family members in postnatal care: Evidence from mother-caregiver dyads in India

**DOI:** 10.1371/journal.pone.0341818

**Published:** 2026-01-27

**Authors:** Pooja Suri, Sahana SD, Shirley Yan, Seema Murthy, Jamie Sewan Johnston

There are errors in the Acknowledgement section. The correct Acknowledgement section is as follows: The authors would like to thank Nikhil Ramnarayan, Abel Desai, Annika Suri, Pradeep Kumar K, Veena J C, Pooja M T, Reha I, Jayasheela D M, Nisha Gupta, Babitha Sharma, Prasad Bogam, Sehj Kashyap, Ramaswamy Premkumar, Shailendra Tomar, Vinayaka A M, Huma Sulaiman, Sareen Kak, Bhanu Pratap Yadav, Tanmay Singh Pathani, and the data collectors who conducted phone surveys from the Noora Health and Yosaid research and implementation teams staff who supported the development of this manuscript, and the program staff. The authors thank Dr. Archana Mishra and the National Health Missions of Karnataka, Madhya Pradesh, Maharashtra, Punjab, for providing support for the study. Most importantly, they express their gratitude to the families who participated in the survey. The authors are grateful for the support of Stanford Center for Health Education Digital Medic initiative, led by Charles Prober and Aarti Porwal. They also thank William Dow, Lia Fernald, and the participants at Fernald Research Group Seminar for their invaluable feedback.
