# Supplementary material for: Investigating the role of family members in postnatal care: Evidence from mother-caregiver dyads in India
Source: PLoS One. 2025 Sep 10;20(9):e0327986. doi: 10.1371/journal.pone.0327986 (PMC12422426; doi:10.1371/journal.pone.0327986)
Supplement: Supplementary file 1 [file pone.0327986.s001.docx]

Table S1. Ty

Caregiver typ
Spouse
Mother
Mother-in-lav

S2. Mental w

I am afraid to
The ASHA/A
I don’t have a
